# Genomic adaptations enabling *Acidithiobacillus* distribution across wide-ranging hot spring temperatures and pHs

**DOI:** 10.1186/s40168-021-01090-1

**Published:** 2021-06-11

**Authors:** Chanenath Sriaporn, Kathleen A. Campbell, Martin J. Van Kranendonk, Kim M. Handley

**Affiliations:** 1 School of Biological Sciences, The University of Auckland, Auckland, New Zealand; 2 School of Environment & Te Ao Mārama – Centre for Fundamental Inquiry, The University of Auckland, Auckland, New Zealand; 3 Australian Centre for Astrobiology & School of Biological, Earth and Environmental Sciences, University of New South Wales, Sydney, Australia

**Keywords:** *Acidithiobacillus*, Genome streamlining, Adaptation, Hot spring, Temperature, pH

## Abstract

**Background:**

Terrestrial hot spring settings span a broad spectrum of physicochemistries. Physicochemical parameters, such as pH and temperature, are key factors influencing differences in microbial composition across diverse geothermal areas. Nonetheless, analysis of hot spring pools from the Taupo Volcanic Zone (TVZ), New Zealand, revealed that some members of the bacterial genus, *Acidithiobacillus*, are prevalent across wide ranges of hot spring pHs and temperatures. To determine the genomic attributes of *Acidithiobacillus* that inhabit such diverse conditions, we assembled the genomes of 19 uncultivated hot spring *Acidithiobacillus* strains from six geothermal areas and compared these to 37 publicly available *Acidithiobacillus* genomes from various habitats.

**Results:**

Analysis of 16S rRNA gene amplicons from 138 samples revealed that *Acidithiobacillus* comprised on average 11.4 ± 16.8% of hot spring prokaryotic communities, with three *Acidithiobacillus* amplicon sequence variants (ASVs) (TVZ_G1, TVZ_G2, TVZ_G3) accounting for > 90% of *Acidithiobacillus* in terms of relative abundance, and occurring in 126 out of 138 samples across wide ranges of temperature (17.5–92.9 °C) and pH (1.0–7.5). We recovered 19 environmental genomes belonging to each of these three ASVs, as well as a fourth related group (TVZ_G4). Based on genome average nucleotide identities, the four groups (TVZ_G1-TVZ_G4) constitute distinct species (ANI < 96.5%) of which three are novel *Acidithiobacillus* species (TVZ_G2-TVZ_G4) and one belongs to *Acidithiobacillus caldus* (TVZ_G1). All four TVZ *Acidithiobacillus* groups were found in hot springs with temperatures above the previously known limit for the genus (up to 40 °C higher), likely due to significantly higher proline and GC contents than other *Acidithiobacillus* species, which are known to increase thermostability. Results also indicate hot spring-associated *Acidithiobacillus* have undergone genome streamlining, likely due to thermal adaptation. Moreover, our data suggest that *Acidithiobacillus* prevalence across varied hot spring pHs is supported by distinct strategies, whereby TVZ_G2-TVZ_G4 regulate pH homeostasis mostly through Na^+^/H^+^ antiporters and proton-efflux ATPases, whereas TVZ_G1 mainly relies on amino acid decarboxylases.

**Conclusions:**

This study provides insights into the distribution of *Acidithiobacillus* species across diverse hot spring physichochemistries and determines genomic features and adaptations that potentially enable *Acidithiobacillus* species to colonize a broad range of temperatures and pHs in geothermal environments.

Video Abstract

**Supplementary Information:**

The online version contains supplementary material available at 10.1186/s40168-021-01090-1.

## Background

*Acidithiobacillus* (formerly *Thiobacillus*) is a gram-negative genus of rod-shaped bacteria that mostly comprises chemolithoautotrophic, obligately acidophilic (optimum pH < 4), and mesophilic or mesothermophilic species [1–3]. They are commonly distributed in acidic and sulfur-rich environments, such as acidic soil and acid mine drainage [4, 5], and recent studies show that they are widely distributed in hot springs [6, 7]. Outside of hot springs, known members of *Acidithiobacillus* have small differences in physiological tolerances to environmental conditions, and all grow within a pH range of 0.5–6.0, and a temperature range of 5–52 °C [3, 8–14]. Of these, *A. caldus*, one of the most studied species in the genus *Acidithiobacillus*, is the only thermoacidophile (first recovered from coal spoils) and has the highest known temperature limit (52 °C) [3, 12, 13, 15, 16]. Other members of the genus, such as *A. ferrooxidans* and *A. thiooxidans*, as well as *A. caldus*, are used in biohydrometallurgy to recover certain metals from sulfide ores due to their ability to oxidize sulfide and solubilize metals, and their preference for acidic environments [10, 11, 17–19]. Although members of *Acidithiobacillus* are best known from highly acidic settings, such as ores during bioleaching and acid mine drainage [4, 20], a search of data from almost one thousand Taupo Volcanic Zone (TVZ) hot springs, in New Zealand, showed that *Acidithiobacillus* are present in springs encompassing a huge range of physicochemistries, from very acidic to alkali pHs (0.6–8.94) and across an extremely wide temperature range (13.9–97.6 °C) [6, 21].

Terrestrial hot spring environments are highly heterogeneous, representing a huge spectrum of physicochemical conditions [6, 22]. Previous studies have suggested that pH and temperature are key drivers influencing microbial composition of hot springs [6, 7]. However, it has been shown that *Acidithiobacillus* are present in numerous hot springs that span a wide range of pHs and temperatures in the TVZ [6, 7, 23]. This suggests that this bacterial genus possesses mechanisms enabling it to inhabit diverse environmental conditions. However, it remains to be determined whether this cosmopolitanism is due to the widespread occurrence of particular *Acidithiobacillus* species or strains across different hot spring environments, reflecting strain or species level metabolic versatility, or whether different hot spring niches harbor phylogenetically distinct *Acidithiobacillus*.

To determine the phylogenetic variation and distribution of *Acidithiobacillus* (clades or genome-inferred species) across broad ranges of hot spring temperatures and pHs, and to elucidate the genomic features underpinning their prevalence, we collected 79 subaqueous sediment samples from various geothermal sites in the TVZ and analyzed these alongside a further 59 subaerial siliceous hot spring deposits (sinters) we previously found to be rich in the *Acidithiobacillus* genus [7]. Sediment-sampled hot spring pHs ranged from 2.0–7.5 and temperatures ranged from 17.5–92.9 °C, while sinter-sampled spring pHs ranged from 1.0–6.6 and temperatures ranged from 24.2–92.9 °C (Table S1). We determined the distribution of *Acidithiobacillus* across these samples and recovered genomes from representative samples with widespread *Acidithiobacillus* 16S rRNA gene amplicon sequence variants (ASVs). Using these genomes, we determined mechanisms of temperature and pH tolerance in the TVZ *Acidithiobacillus* groups and compared these and other genomic attributes to *Acidithiobacillus* from other environments. Results show that the TVZ *Acidithiobacillus* have distinct genomic features that potentially allow them to inhabit broad ranges of temperature and pH.

## Materials and methods

### Sample collection and physicochemical measurements

Fifty-nine sinter samples (with spatial replicates included) were collected from 10 hot springs across five geothermal areas (Orange Spring, Te Kopia, Parariki Stream, Rotokawa, and Tikitere) within the TVZ (Fig. 1 from [7]) in April 2018, as described in Sriaporn et al. [7]. Seventy-nine additional sediment samples (with spatial replicates included) were collected from 12 hot springs across four geothermal areas (Parariki Stream, Rotokawa, Tikitere, and Wai-O-Tapu) within the TVZ in February and November 2019. Sediment was collected from 0 to approximately a few centimeters below the water-sediment interface using sterile spatulas, and samples were placed into sterile 50 mL centrifuge tubes and transported on dry ice, before storage at − 80 °C. Hot spring pH and temperature measurements from the April 2018 sinter sampling trip were measured using portable sension™ 156 Multiparameter Meter (Hach Company, USA) and COMARK Evolution IV9001 temperature probes (COMARK, UK) [7], and pH and temperatures from the sediment sampling trips (February and November 2019) were measured using a WTW 330i handheld meter (WTW GmbH, Germany).

**Fig. 1:**
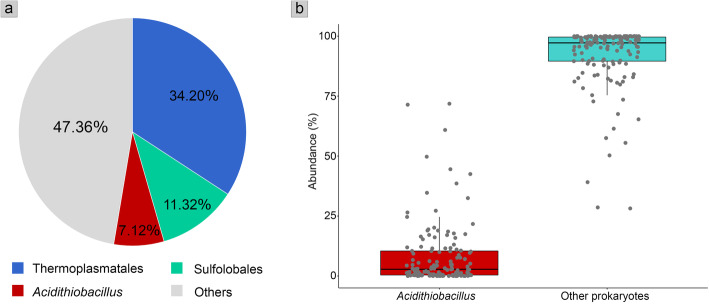
Plots showing the proportion of *Acidithiobacillus* ASVs observed in microbial communities across the TVZ hot spring sites. **a** Total relative abundance (%) of prokaryotic communities summed across all samples collected across six geothermal areas in the TVZ. **b** Boxplots showing total relative abundance of *Acidithiobacillus* versus other prokaryotes in each sample. Note that there are 12 samples that did not harbor *Acidithiobacillus* (shown at 0% in the left box plot, and 100% in the right box plot)

### DNA extraction, amplicon, and metagenomic sequencing

DNA extraction was performed using the DNeasy PowerSoil® Kit (Qiagen, USA) following the manufacturer’s instructions, using 0.25–0.35 g of sinter or sediment per sample (with a no-sample negative control included). DNA quality and quantity were checked via gel electrophoresis, a NanoPhotometer (Implen, Germany), and Qubit 3.0 fluorometric quantitation (Invitrogen, USA). 16S rRNA gene amplification was performed in triplicate using 515′F/926′R primers, and 2 × 250 bp paired-end Illumina MiSeq sequencing was performed with the MiSeq Reagent Kit V2, as previously described [7], at Auckland Genomics (University of Auckland). To explore the genomic features of the TVZ *Acidithiobacillus*, 4 representative sinter and 18 representative sediment samples were selected for whole genome sequencing (WGS). Five to seven g of DNA was extracted per sample using the DNeasy PowerMax Soil Kit® (Qiagen, USA). Due to low DNA concentrations, multiple extractions were performed for each sample, and DNA was concentrated and cleaned by ethanol precipitation using 2× absolute ethanol, 3 M sodium acetate, and 20 μl/μg glycogen. Samples were then incubated at − 80 °C overnight before DNA pellets were washed with 70% ethanol and resuspended in 25 μl of nuclease-free water. Final DNA quality and quantity were checked using an Implen NanoPhotometer, Qubit 3.0 fluorometric quantitation with the Qubit™ dsDNA HS Assay Kit, and gel electrophoresis. WGS sequencing was performed at the Otago Genomics Facility (University of Otago, Dunedin, New Zealand). Thruplex DNA-Seq libraries (Takara Bio USA, Inc, USA) were prepared with 500–600 bp insert sizes and sequenced using the Illumina HiSeq 2500 platform with the HiSeq Rapid SBS Kit V2, yielding 2 × 250 bp reads, for the 4 sinter samples, and the HiSeq 2500 SBS Kit V4, yielding 2 × 125 bp reads, for the 18 sediment samples.

### Amplicon data analysis

QIIME2 (version 2019.10) was used to process demultiplexed amplicon sequences by read-joining, quality filtering (Q score cutoff of 25) and denoising (with singletons removed), and to generate an ASV table [24–27]. Taxonomy was assigned by using the SILVA database version 132 [28–30]. R (version 3.4.4) with RStudio software (version 3.4.1), and R package ggplot2 (version 3.0.0) were used to visualize data [31–33].

### Metagenomic sequence processing and genome assembly

Quality filtering and trimming of raw metagenomic sequences were undertaken using Sickle (version 1.33) with a minimum quality score threshold of 30 and a minimum retained read length of 80 bp, and FastQC version 0.11.9 was used to check quality [34, 35]. After trimming 86.5–92.2% of reads per sample were retained. Paired-end trimmed reads from each sample were then assembled separately using SPAdes version 3.11.1 with –meta -m 900 -t 16 -k 41,61,81,101,127 parameters [36]. To determine differential coverage for genome binning, reads were mapped to contigs using Bowtie version 1.2.0 with the following parameters: --phred33-quals -n 1 -l 111 --minins 100 --maxins 600 --best [37]. Contigs were binned using MetaBAT version 2.12.1 [38], MaxBin version 2.2.4 [39], and CONCOCT version 0.4.1 [40], utilizing differential coverage and tetranucleotide frequencies. The resulting bins (metagenome-assembled genomes, MAGs) were compared, and representatives were selected using DAS Tool version 1.1.1 [41]. Bin refinement was performed using VizBin with differential coverage incorporated [42]. CheckM version 1.0.12 [43] was used to estimate refined bin completeness and contamination. Estimated genome size was calculated following Castelle et al. [44]. Bins shared across the 4 sinter and 18 sediment sample assemblies were then dereplicated using dRep version 1.4.3 based on the default 99% similarity threshold to generate a set of unique representative bins [45].

### Gene prediction, annotation, and genome analyses

Gene prediction was performed using Prodigal version 2.6.3 with -p meta mode [46]. Predicted coding DNA sequences (CDS) were annotated against the UniRef100 [47], UniProt [48], KEGG [49], Pfam [50], and TIGRFAM [51] databases using USEARCH version 9.0.2132 [52] with an e-value cutoff of 0.001. Prokka version 1.13.4 was used to predict tRNAs, tmRNAs, CRISPRs, and non-coding RNA [53]. Taxonomic classification was assigned based on core marker genes using GTDB-Tk version 0.2.1 [54]. The genome sequences of nine *Acidithiobacillus* type strains (*A. caldus* ATCC 51756, *A. caldus* SM-1, *A. thiooxidans* ATCC 19377, *A. ferrooxidans* ATCC 23270, *A. ferrivorans* SS3, *A. albertensis* DSM 14366, *A. ferridurans* JCM 18981, *A. ferrianus* MG, and *A. sulfuriphilus* CJ-2) and one unclassified species (*Acidithiobacillus* sp. UBA2486), obtained from the NCBI GenBank database, were included in these gene prediction and annotation steps for comparison with the studied TVZ *Acidithiobacillus* genomes.

To determine whether genomes belonged to the same species, the TVZ *Acidithiobacillus* bins and reference genomes were compared by calculating pairwise average nucleotide identity (ANI) values via the average nucleotide identity calculator [55] based on the approach described by Goris et al. [56] with 96.5% threshold for determining the same species [57, 58]. The Genome-to-Genome Distance Calculator (GGDC) was used to approximate in silico DNA-DNA hybridization (DDH) values to determine the likelihood that any two genomes belong to the same species using a 70% threshold [59, 60]. To examine mutation events discriminating subspecies genome clusters, GSAlign was used to detect substitutions, insertions, and deletions between genomes [61]. In addition, BLAST was used to determine the identity of 16S rRNA genes among the TVZ and other *Acidithiobacillus* species using the > 99% species threshold [62].

To further compare *Acidithiobacillus* attributes, we predicted genome minimum generation times and optimal growth temperatures using GrowthPred [63]. Genome replication rates at the time of sampling were estimated using the index of replication (iRep) [64]. Protein paralogs were determined using CD-HIT [65]. Proteins that were at least 30% identical over at least 50% of the longest sequence length were considered paralogs [66]. Genome GC contents were derived from CheckM output, and proline content was calculated from predicted protein sequences using an in-house python script [67].

To explore the genomic features of *Acidithiobacillus* from a wider set of environments, an additional 27 *Acidithiobacillus* genomes from Genome Taxonomy Database (GTDB) [68] were included for comparative analyses of genome size, optimal growth temperature, paralogs, GC contents, and proline composition (Table S2). Correlation plots between genomic features were generated using RStudio (package ggplot2), and Pearson’s correlation coefficients and *t*-distribution tables (*df* = *n* − 1) were used to determine the correlation coefficients and significance of correlations, respectively (via package ggpubr). A *T*-test was used to determine if any significant differences were present between two groups of genomes.

### Extraction and reconstruction of partial and near full-length 16S rRNA gene sequences

16S rRNA gene sequences were reconstructed from each quality-filtered WGS sample using EMIRGE version 0.61.1 with a 97% clustering threshold [69]. Taxonomy was assigned using USEARCH version 11.0.667 (SINTAX algorithm) by searching sequences against the SILVA SSU non-redundant database version 132 [29, 52]. In addition, MeTaxa2 [70] was used to extract 16S rRNA genes from MAGs. In order to match independently reconstructed 16S rRNA sequences (EMIRGE), MAG-extracted 16S rRNA genes (MeTaxa2), and ASVs, relative abundance correlations were undertaken using RStudio (with package ggplot2). Pearson’s correlation coefficients and *t*-distribution tables (*df* = *n −* 1) were used to determine the correlation coefficient and the statistical significance, respectively (via package ggpubr) [71]. Sequences derived from the three approaches were aligned with Geneious version 11.1.2 using MUSCLE [72, 73].

### *Acidithiobacillus* phylogenetic tree

A concatenated core gene phylogenetic tree was constructed using the TVZ *Acidithiobacillus* MAGs and the 10 *Acidithiobacillus* reference genomes obtained from the GenBank and GTDB databases [68]. Sequences were aligned using MUSCLE with Geneious [72, 73], and PhyML 3.0 was used to build and visualize a maximum-likelihood phylogenetic tree with 100 times bootstrapping [74].

## Results and discussion

### Hot spring characteristics

Samples were collected from sulfur-rich hot springs, which are common in the TVZ, and which spanned a broad range of water temperatures (17.5–92.9 °C) to recover mesophilic to hyperthermophilic communities, and pHs (1.0–7.5) to sample acidophilic to neutrophilic communities (Table 1, Fig. S1). Samples comprised subaerial digitate sinter and subaqueous sediments. The digitate sinters are stromatolitic siliceous deposits with protrusive features that form around hot spring margins or in shallow outflow channels just above water level (reaching approximately 1 cm above the air-water interface) [7], due to the deposition of silica from evaporative wicking of spring water [75]. In contrast, sediments (i.e., hot spring mud and unconsolidated coarser geothermal-influenced stream material) form within hot springs when underground gases react with rocks to produce clay, or are detrital (i.e., broken up pieces of sinter, surrounding rocks, and organic matter) [22], and for this study were located a few millimeters to centimeters below the water’s surface. Digitate sinter and sediment from the same pool were connected by the thermal fluids. Subaerial sinter samples were taken from five geothermal areas, which are ~ 1 to 65 km apart, with a proximal water temperature range of 24.2–92.9 °C and pHs of 1.0–6.6 (Table 1, Fig. S1). Subaqueous sediment samples were collected from four geothermal areas (~ 1 to 65 km apart) with a direct water temperature range of 17.5–92.9 °C and pH range of 2.0–7.5. It is worth noting that as the temperature and pH measurements in this study were based on hot spring fluids, they may not represent actual values for the subaerial sinter samples, but apply directly to sediment samples, which are submerged within the spring water (Table S1).

**Table 1:** Site, pH and temperature ranges, and water chemistry of studied geothermal settings, including collected sample type and studied data type

Site	pH range	Temperature range (°C)	Water chemistry	Sample type	Data type
Tikitere	3.60–7.50	17.5–73.8	Acid-sulfate-bicarbonate	Sinter, sediment	WGS, 16S
Parariki Stream	1.00–2.14	33.4–77.0	Acid-sulfate-chloride	Sinter, sediment	WGS, 16S
Rotokawa	1.73–2.94	22.7–80.1	Acid-sulfate-chloride	Sinter, sediment	WGS, 16S
Te Kopia	2.14–2.22	43.6–92.9	Acid-sulfate	Sinter	WGS, 16S
Orange Spring	1.80–2.65	35.4–91.5	Acid-sulfate	Sinter	16S
Wai-O-Tapu	2.51–5.70	32.4–92.9	Acid-sulfate-chloride	Sediment	WGS, 16S

WGS = Whole genome sequencing, 16S = 16S rRNA gene amplicon sequencing

### Distribution of TVZ *Acidithiobacillus* across wide ranges of temperature and pH

16S rRNA gene amplicon sequencing of 138 subaerial sinter and subaqueous sediment samples produced a total of 28,204 ASV features (with 1,344,820 ASV counts). These were classified into 49 phyla, and the overall community distribution is shown in Figure S2. Of these, 1195 ASV features (95,787 ASV counts) were from genus *Acidithiobacillus* (order Acidithiobacillales), which comprised 7.1% in total (or on average 11.4 ± 16.8%), of prokaryotic communities across all of the hot springs sampled for this study (Fig. 1a, b). It was also the third most abundant taxon, following the archaeal orders Thermoplasmatales and Sulfolobales, and hence was the most abundant bacterial genus (Fig. 1). This corresponds well with our previous work and that of other studies, confirming the high relative abundance of *Acidithiobacillus* in sulfuric hot spring environments (sediment, sinter, soil, and water) in both New Zealand [6, 7, 23, 76] and elsewhere [77, 78]. One explanation for *Acidithiobacillus* predominance in these settings is the various genes for sulfur metabolism they characteristically possess, which enable them to oxidize sulfur, thiosulfate, sulfide, and sulfite in geothermal environments [13, 79].

**Fig. 2:**
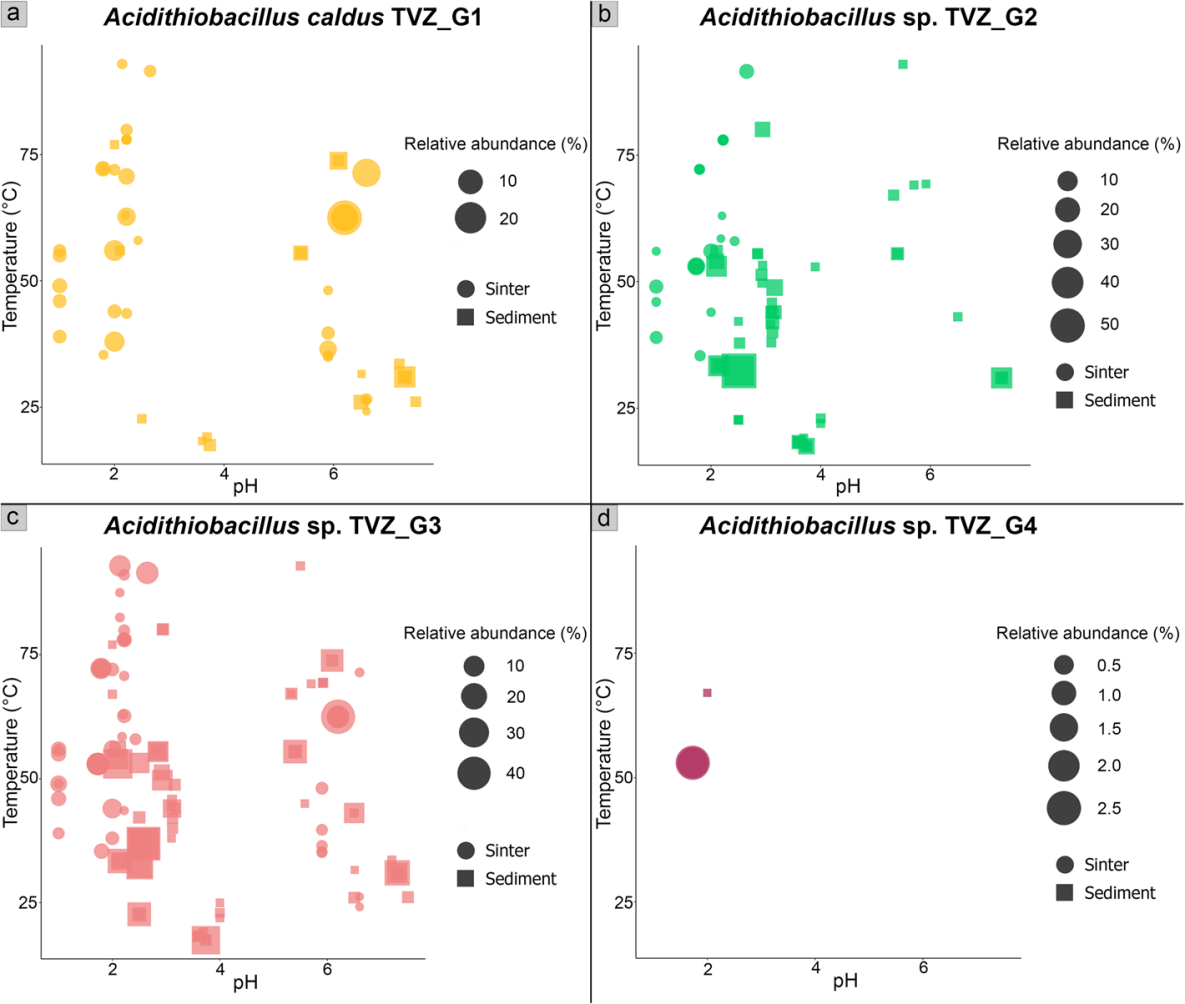
Scatter plots showing relative abundances and prevalence of the four TVZ hot spring *Acidithiobacillus* ASVs that correspond with recovered genomes of **a** *A. caldus* TVZ_G1; **b** *Acidithiobacillus* sp. TVZ_G2; **c** *Acidithiobacillus* sp. TVZ_G3; and **d** *Acidithiobacillus* TVZ_G4. Bubble size represents the relative abundance of each ASV per sample. The number of bubbles represents the prevalence of each *Acidithiobacillus* ASV

**Fig. 3:**
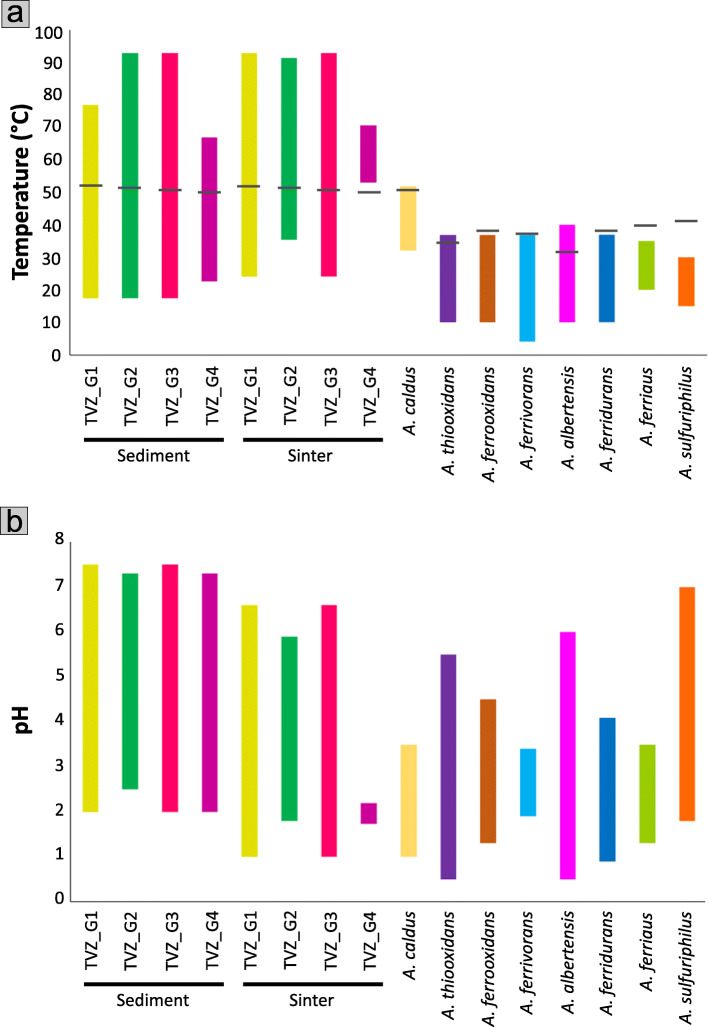
Plots showing the physicochemical ranges of TVZ *Acidithiobacillus* and those known for other *Acidithiobacillus*. Ranges are given for **a** temperature (with black bars indicating optimal growth temperatures calculated by GrowthPred) and **b** pH. Ranges shown for subaerial sinters are from the corresponding hot spring waters from which the digitate sinters form. All temperature and pH ranges shown for the TVZ species represent the springs from where they were found, while those for other *Acidithiobacillus* are from growth experiment characterizations

Overall, we recovered more than 1000 *Acidithiobacillus* ASVs from 126 out of 138 (91.3% of) samples, spanning a temperature range of 17.5–92.9 °C for sediment samples and 24.2–92.9 °C for sinter samples, and a pH range of 2.0–7.5 for sediment samples and 1.0–6.6 for sinter samples (Fig. S1, Table S1, S3). Of these, just three ASVs constituted more than 90% of the total *Acidithiobacillus* population and were detected across 126 of the samples, suggestive of both their abundance and prevalence across a wide range of temperatures and pHs in geothermal environments (Fig. 2a–c, Fig. S3, Table S3). These are amongst the widest physicochemical ranges recorded for *Acidithiobacillus* (Fig. 3a, b) and are similar to findings from the TVZ 1000springs project [21], in which *Acidithiobacillus* was found in springs with pHs 0.6–8.94 and temperatures of 13.9–97.6 °C. However, this earlier project did not classify to species level. The only currently known thermoacidophilic *Acidithiobacillus* species, *A. caldus* (type strain ATCC 51756), has a known upper temperature limit of 52 °C (as characterized by growth experiments), which is the highest for this genus [3]. Nevertheless, more than 50% of our samples that harbored *Acidithiobacillus* were from sites with temperatures higher than 52 °C (Fig. S1, Table S1). These results indicate that the TVZ *Acidithiobacillus* may survive in conditions that exceed the formerly known upper pH limit of 6.0, which was recorded for *A. albertensis* (originally isolated from acidic soil) [8].

Characterization and growth experiments of TVZ *Acidithiobacillus* are needed to confirm the expansion of the upper physicochemical limits of *Acidithiobacillus* to include high temperature and circumneutral pH environments. Nevertheless, while the possibility of exogenous transport cannot be excluded, this seems unlikely given the high relative abundance of *Acidithiobacillus* in the studied springs, implying that these organisms are not transient. In addition, while all sampling sites were in the TVZ, each site was isolated and distant (1–65 km apart). Moreover, alpha diversity trends are consistent with expectations based on hot spring physicochemistry [7]. Shannon indices show that Tikitere had the most diverse communities overall, including the most diverse *Acidithiobacillus* population, likely due to the near-neutral pHs and relatively moderate temperatures (Fig. S4, Table S1). In contrast, Parariki Stream, Rotokawa, Te Kopia, and Wai-O-Tapu shared the lowest overall community diversity. Locations sampled at each of these sites were exclusively or predominately low in pH. Accordingly, results showed that, for *Acidithiobacillus* populations, the lowest diversity was sampled at Te Kopia. This is likely due to the very extreme temperatures and pHs (up to 93 °C and with pHs of 2.1–2.2), which are amongst the highest and lowest, respectively, in this study, and are potentially at or near *Acidithiobacillus* limits in these systems.

### Phylogeny of TVZ *Acidithiobacillus* and the prevalence and predominance of distinct populations

To determine the phylogeny and genomic features that potentially explain the broad temperature and pH tolerances of the three abundant and prevalent *Acidithiobacillus* ASVs, we reconstructed 19 *Acidithiobacillus* MAGs from 22 representative samples (4 sinters and 18 sediments). All 19 MAGs were estimated to be largely or near-complete (78.2–99.4%; Table S2) with low contamination (0–2.59%; Table S2). Genomes were clustered into four groups based on an average nucleic acid similarity threshold of 99%. A partial 16S rRNA sequence was extracted from each representative MAG within each of the four groups. Additionally, EMIRGE recovered 232 near full-length 16S rRNA gene sequences from the 22 WGS samples, and three of these sequences were identical to three out of four *Acidithiobacillus* MAG-derived and three abundant and prevalent *Acidithiobacillus* ASVs based on sequence alignments (Fig. S5). The extra fourth MAG-derived 16S rRNA sequence was identical to a rarer *Acidithiobacillus* ASV (Table S3), which accounted for 0.4 ± 5.9% average (or 0.025% total) relative abundance. We designate these four *Acidithiobacillus* groups: TVZ_G1, TVZ_G2, TVZ_G3, and TVZ_G4 (Fig. 2a–d, Fig. S3, Table S3). Results also indicate a broadly positive correlation between the relative abundances of 16S rRNA sequences (amplicon and EMIRGE) and the genome coverage of the four representative MAGs (Fig. S6).

**Fig. 4:**
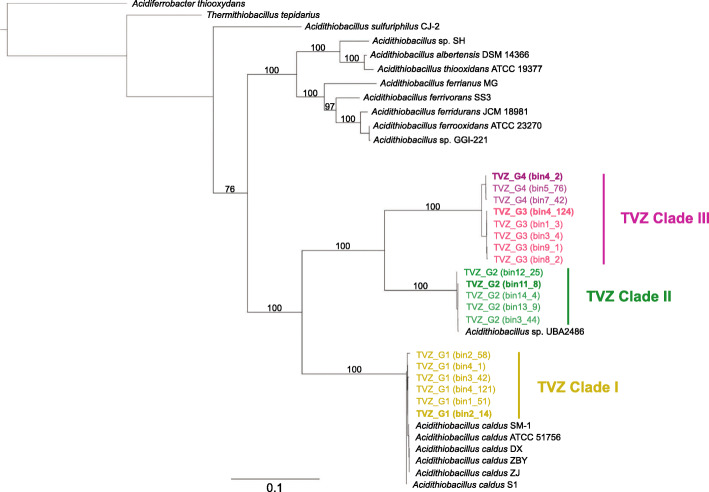
A maximum-likelihood concatenated core gene phylogenetic tree with 100 times bootstrapping of TVZ and other *Acidithiobacillus* species. Bold script indicates the representative MAG of each species (TVZ_G2-4) or subspecies (TVZ_G1) level cluster. *Thermithiobacillus tepidarius* from another family and *Acidiferrobacter thiooxydans* from another class are used as outgroups

EMIRGE-derived 16S rRNA gene sequences of TVZ *Acidithiobacillus* shared < 99% identity with other reference *Acidithiobacillus* 16S rRNA gene sequences (Table S4), indicating their novelty [62]. Pairwise in silico DDH and ANI comparisons also showed that representative MAGs within the four *Acidithiobacillus* groups shared values below the in silico DDH (70%) and species delineation (96.5%) cut-offs [57–60] (Tables S5-6), suggesting each are a distinct species. In comparison, MAGs within each group represent conspecific populations, on the basis of DDH and ANI values of 91.1–100% and 98.8–100%, respectively—above the species threshold. Results indicate that three out of four of the groups (TVZ_G2-TVZ_G4) are novel species, while TVZ_G1 belongs to *A. caldus*. TVZ_G1 shares an in silico DDH value of 96% and ANI of 99.6% (based on the representative genome) with the *A. caldus* type strain ATCC51756 (Table S5-6). Accordingly, a concatenated core gene phylogenetic tree shows the four TVZ groups formed three distinct clades within the genus *Acidithiobacillus* (Fig. 4), with closely related TVZ_G3 and TVZ_G4 populations representing sub-clades of Clade III, and the six MAGS comprising TVZ_G1 representing a discrete clade within *A. caldus* (Clade I). In addition, while TVZ_G2 is unrelated to any formerly designated and cultivated species, its five MAGs clustered with one uncultivated bacterium from a Taiwanese hot spring, *Acidithiobacillus* sp. UBA2486 (Clade II, Fig. 4), which ANI and in silico DDH values indicate are the same species (Table S5-6).

Of the three most abundant *Acidithiobacillus* populations, *A. caldus* TVZ_G1 was the least abundant (10.6 ± 2.7% ASV relative abundance) (Fig. 2a–c, Fig. S3, Table S3). This is contrary to previous studies, where it has been suggested that the dominant *Acidithiobacillus* species in geothermal settings is *A. caldus* [23, 78], although such classifications were based on relatively low percent identity matches (i.e., 97% partial 16S gene sequence identity). Instead, we found that the novel TVZ_G3 population was the most abundant overall, representing on average 58.8 ± 1.3% of *the Acidithiobacillus* population (or 41.5 ± 22.4% based on genome coverage) (Fig. 2a–d, Fig. S3, S7-8, Table S3), while its close relative, TVZ_G4, comprised only 0.4 ± 5.9% of *Acidithiobacillus* ASVs (or 5.3 ± 2.2% genome abundance). Of these, TVZ_G3 was also the most prevalent group, being distributed across 86.2% of samples based on the ASV (or 45.5% of the WGS subset of samples). This was followed by TVZ_G2 (52.2% ASV prevalence or 45.5% of the WGS subset) and TVZ_G1 (40.6% ASV prevalence or 36.4% of the WGS subset) (Fig. 2a–d, Fig. S3, S7-8, Table S3). In addition to its low abundance, TVZ_G4 was also the least prevalent among the four as shown by amplicon data (2.2%) and genome coverage (31.8%) (Fig. 2d, Fig. S3, S8).

Our study shows that three of the TVZ *Acidithiobacillus* species were present across diverse and spatially distinct hot spring environments and were therefore unconstrained by geographical isolation. However, only two genomes within TVZ_G2 were found to share 100% ANI based on their alignable fractions with the group reference MAG (Table S5). To further explore genetic diversity within the TVZ *Acidithiobacillus* species, we determined substitutions (single-nucleotide variants, SNVs) and insertions and deletions (indels). Results indicated a predominance of substitutions, and relatively few indels, when comparing MAG consensus sequences with the representative per group (Fig. S9). MAGs belonging to *A. caldus* TVZ_G1 and *Acidithiobacillus* TVZ_G2 exhibited the least intra-group genomic variation, with 0.005–0.15% SNVs and 0–0.002% indels (74 to 1746 substitutions and 0 to 26 indels per Mbp). The highest amount of genetic variation was detected in TVZ_G3 and TVZ_G4, with up to and 0.003% and 0.004% indels (44 and 45 events per Mbp), respectively, and up to 0.5% and 0.9% SNVs (8087 and 10,722 of substitutions per Mbp), respectively.

These intraspecies genetic diversities are small, compared to other studies where 2.6–9.7% SNVs were observed among non-hot spring *A. caldus* strains, and 2.5–3.5% single-nucleotide polymorphisms among *A. ferrooxidans* strains [80, 81]. Nevertheless, the upper values for single-nucleotide differences detected among TVZ populations are above Illumina sequencing error rates determined for metagenomics (0.04–0.12% for SNVs and 0.00028–0.00051% for indels) [82], and support for the consensus sequences is afforded by overall high genome coverages (on average 37.4 ± 45.7 × coverage) (Table S2), which indicates variations represent real biological differences. Genetic variations among MAGs recovered from unique TVZ hot springs between 1 km and 65 km apart suggests that successful colonization of these diverse habitats by cosmopolitan *Acidithiobacillus* species is achieved via population-level diversification, either due to environmental selection controlled by the unique chemical conditions of each hot spring or genetic drift and dispersal limitation [83, 84]. This is exemplified by habitat-differentiated ecotypes of the pelagic cosmopolitan genus *Prochlorococcus* [85]. Similarly, distinct clades of *Sulfolobus* have been found in remote hot springs [86], indicating a high degree of genetic variation driven by spatial separation.

### GC contents correspond with optimal growth temperatures in *Acidithiobacillus*

Predicted optimal growth temperatures for the TVZ *Acidithiobacillus* species and other *Acidithiobacillus* type strains by GrowthPred are shown in Fig. 3a, along with the actual temperature ranges across which they were found. Results show that for *Acidithiobacillus* isolates GrowthPred predicted optimal growth temperatures near or at the reported upper temperature limits of these organisms, as expected based on the master reaction model for temperature growth curves [87, 88]. On the other hand, for TVZ populations, GrowthPred predicted optimal growth temperatures to typically fall in the middle of the temperature ranges at which they were detected, possibly due to different tolerances within populations.

**Fig. 5:**
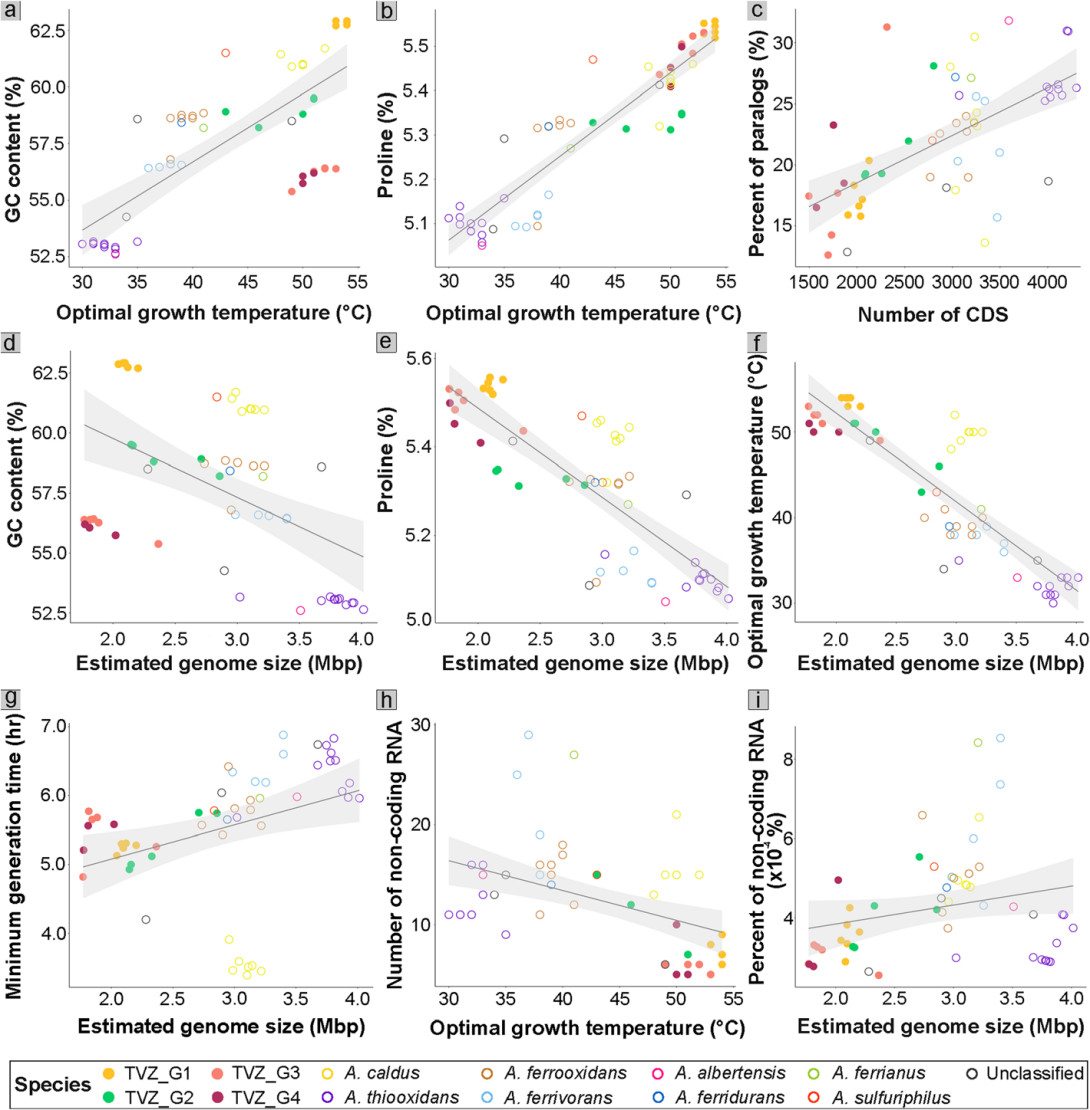
Scatter plots of the TVZ and other *Acidithiobacillus* species. Plots **a**–**c** show positive correlations between **a** GC content (%) and optimal growth temperature (°C) (*R* = 0.75, *p* = 3.0 × 10^−11^); **b** predicted proline contents (%) and optimal growth temperature (°C) (*R* = 0.92, *p* < 2.2 × 10^−16^); and **c** percentage of paralogous proteins and coding sequences (CDS) (*R* = 0.63, *p* = 1.7 × 10^−7^). Plots **d**–**f** show negative correlations between **d** GC content (%) and estimated genome size (Mbp) (*R* = − 0.51, *p* = 7.0 × 10^−5^); **e** predicted proline content (%) and estimated genome size (Mbp) (*R* = − 0.81, *p* = 3.4 × 10^−14^); and **f** optimal growth temperature (°C) and estimated genome size (Mbp) (*R* = − 0.86, *p* < 2.2 × 10^−16^). **g** Shows a positive correlation between minimum generation time (hr) and estimated genome size (Mbp), with reference *A. caldus* and the Taiwanese *Acidithiobacillus* (UBA2486) as outliers (*R* = 0.36, *p* = 0.008). **h** Shows a negative correlation between number of non-coding RNA and optimal growth temperature (°C) (*R* = − 0.45, *p* = 0.00044). **i** Shows a positive correlation between percent of non-coding RNA and estimated genome size (Mbp) (*R* = 0.24, *p* = 0.08). Optimal growth temperatures were derived from GrowthPred calculation. The nearest (co-clustering) unclassified *Acidithiobacillus* species (black rimmed-circle) to TVZ_G2 is *Acidithiobacillus* sp. UBA2486 in all plots. Pearson’s correlation coefficients and *t*-distribution tables were used to determine the correlation coefficients and the significances, respectively, as described in the methods

In order to determine whether GC content contributes to heat tolerance, we examined the compositions of GC in the TVZ *Acidithiobacillus* and reference genomes. Data show that the TVZ *Acidithiobacillus* groups have an average GC content of 59.0 ± 2.9%, whereas the other 37 reference *Acidithiobacillus* have an average GC content of 56.9 ± 3.2% (Fig. 5a, d). Interestingly, *A. caldus* TVZ_G1 bins alone have average GC contents of 62.8 ± 0.1%. These contents are significantly higher (*p* = 1.2 × 10^-13^) than all other known *Acidithiobacillus* (GC content data retrieved from GTDB and also recalculated by CheckM), of which the GC contents range from 52.6 to 61.7% (Table S2). Even when compared to other strains of *A. caldus*, we found that the TVZ_G1 clade still had a significantly higher GC content (*p* = 2.27 × 10^-6^). Although some studies have reported that *A. caldus* DSM 8584 (or ATCC 51756) contains 63.9% GC [2, 3], this was based on only the 16S rRNA gene sequences, while the whole genome GC content was instead shown to be 61.7% [12].

As it has been debated whether GC content and optimal growth temperature are correlated in prokaryotes [89–92], we determined the relationship between the GC content of taxa and their preferred growth temperatures in order to verify this. Our results indicate that *Acidithiobacillus* GC contents and optimal growth temperatures, as predicted by GrowthPred, are positively correlated (*R* = 0.75, *p* = 3.0 × 10^−11^; Pearson’s correlation coefficient) (Fig. 5a). This suggests higher GC contents might contribute to heat tolerance in *Acidithiobacillus* from the TVZ and elsewhere. Higher thermostability is a well-known characteristic of GC-rich DNA, owing to the greater thermostability imparted by the three hydrogen bonds binding GC, in contrast to the two hydrogen bonds binding AT pairs [90, 92]. In addition, the stacking arrangement of adjacent bases is another important factor contributing to the thermal stability of DNA, as there is more favorable stacking energy for GpC/CpG pairs than for ApT/TpA pairs, such that genomes consisting of more GpC/CpG islands tend to be more thermostable than those that have fewer islands [93].

It is worth noting that GrowthPred calculates an organism’s optimal growth temperature from the frequency of a set of amino acids (IVYWREL), which is the only set (out of all amino acids) that was found to be remarkably positively correlated with optimal growth temperature [94]. The predicted optimal growth temperatures could therefore be influenced by GC-rich codons, associated with five out of seven of this set, which account for more than 40% of GC-rich codons, such as GUC and GUG (for Val, V), UGG (for Trp, W), CGC and CGG (for Arg, R), GAG (for Glu, E), and CUC and CUG (for Leu, L). Nevertheless, positive correlations between GC content and optimal growth temperatures in the TVZ *Acidithiobacillus* are reinforced by the high hot spring temperatures in which these species were detected (Fig. 2a–d).

### High proline composition contributes to heat tolerance in TVZ *Acidithiobacillus*

To examine heat tolerance capability, we determined the correlation between proline content and optimal growth temperature of the TVZ *Acidithiobacillus* groups*.* Results indicated that the TVZ *Acidithiobacillus* genomes, along with the Taiwanese hot spring strain UBA2486, have significantly higher proportions of codons encoding proline than other *Acidithiobacillus* (5.5% ± 0.1 versus 5.2% ± 0.1, *p* = 3.8 × 10^-10^) (Table S2). The proline-coding contents are also positively correlated with optimal growth temperature (*R* = 0.92, *p* < 2.2 × 10^−16^) (Fig. 5b). Notably, proline is not one of the amino acids used by GrowthPred to calculate optimal growth temperature. Protein thermostabilization by proline substitutions is a strategy thermophiles use to cope with high-temperature environments by reducing the conformational degrees of freedom in the main polypeptide chain [95, 96]. The structure of proline is more similar to *α*-imino acid, rather than *α*-amino acid, such that its side chain is folded back to form a peptide bond with nitrogen, which results in rigid constraints on the N-C *α*-rotation. Alongside higher GC contents, higher proline compositions might explain the widespread occurrence of TVZ *Acidithiobacillus* in extremely hot environments that exceed the temperature limits of many other *Acidithiobacillus*.

In addition, we explored differences in heat-shock proteins (HSPs) in the TVZ genomes compared to other reference *Acidithiobacillus* species; however, no difference in HSP abundances (copies per genome) was observed between these groups. We believe the reason for this is that HSPs are designed initially for upshifts/downshifts in temperature. As the TVZ strains were found in high-temperature springs, this suggests adaptation to high temperatures, and no noticeable difference in HSP abundances. However, differences in expression cannot be excluded.

### Evidence of genome streamlining in the TVZ *Acidithiobacillus*

We compared the estimated genome sizes of TVZ and other *Acidithiobacillus* and found the TVZ hot spring groups and the Taiwanese hot spring strain (UBA2486) had considerably smaller genome sizes on average (2.1 ± 0.3 Mbp) compared to those of the reference genomes (3.3 ± 0.4 Mbp, *p* = 3.2 × 10^−12^) (Fig. 5d–g, i). Notably, the genome sizes of the TVZ *A. caldus* clade were also much smaller than other *A. caldus* strains (2.1 ± 0.1 Mbp versus 3.1 ± 0.1 Mbp, *p* = 1.83 × 10^-10^) that are not typically associated with hot spring environments. The smaller estimated genome sizes, particularly for *Acidithiobacillus* spp. TVZ_G3 and TVZ_G4, are indicative of genome streamlining. During streamlining, prokaryotes minimize complexity in their cells in order to make the most use of resources required for replication and, thus, increase fitness [97]. In addition, despite the geographical distance, *Acidithiobacillus* sp. UBA2486, which was recovered from a hot spring environment in Taiwan (temperature = 50–85 °C and pH = 2.5) [98], shares a similar estimated genome size (along with other studied genomic attributes) with the TVZ *Acidithiobacillus* (Fig. 5a–i). Results are in agreement with prior research demonstrating that prokaryotes adapted to high temperatures (in this case, hot springs) tend to have smaller genome sizes, alongside lower proportions of non-coding RNA, suggestive of streamlining (Fig. 5f, h) [99]. Accordingly, we also found broadly lower proportions of non-coding RNA to estimated genome size in TVZ *Acidithiobacillus* spp. (Fig. 5i).

To further explore the link between adaptation to high temperatures and genome reduction in hot spring *Acidithiobacillus*, we plotted estimated genome size against predicted optimal growth temperature. Results illustrate a strong negative correlation between optimal growth temperature and increasing estimated genome size (*R* = − 0.86, *p* < 2.2 × 10^−16^; Pearson’s correlation coefficient) (Fig. 5f). Both smaller genome sizes and higher optimal growth temperatures can underpin faster genome replication rates [97, 99]. Consistent with this, we found that predicted genome replication rates were highest for the smaller hot spring *Acidithiobacillus* genomes, and minimum generation times were lower than for most other *Acidithiobacillus* (Fig. 5g, Table S2). High predicted replication rates may explain the overall high relative abundance of the TVZ *Acidithiobacillus* across the hot spring environments tested. In addition, the identified ranges of iRep values are consistent with those from other studies of actively growing communities [64, 100], implying that these organisms were active in the environment at the time of sampling.

Results further indicate that estimated genome size in *Acidithiobacillus* is negatively correlated with GC content and proline composition (*R* = − 0.51, *p* = 7.0 × 10^−5^ and *R* = − 0.81, *p* = 3.4 × 10^−14^; Pearson’s correlation coefficient) (Fig. 5d, e), even though GC content has been shown elsewhere to be positively correlated with genome size in prokaryotes [101, 102]. Although *A. caldus* TVZ_G1 genomes, in particular, have a relatively high GC content, this contradiction is likely due to the importance of high GC composition for thermotolerance. It is possible, therefore, that TVZ_G1 underwent genome reduction, resulting in smaller genome sizes than other *A. caldus*, yet preserved high GC contents as an adaptive strategy for high-temperature environments.

Finally, streamlined prokaryotes also tend to have a lower fraction of paralogs compared to CDS [57, 84]. We determined the amount of paralogous predicted protein sequences in *Acidithiobacillus* genomes and found that the TVZ *Acidithiobacillus* (along with UBA2486) have lower proportions of paralogous predicted protein sequences than other *Acidithiobacillus* genomes (on average 18.8% ± 4.5 versus 24.0% ± 4.2, *p* = 9.6 × 10^−5^) (Fig. 5c). Our results also show that the percent of paralogs is correlated with CDS (*R* = 0.63, *p* = 1.7 × 10^−7^; Pearson’s correlation coefficient) and genome size (*R* = 0.24, *p* = 0.008; Pearson’s correlation coefficient), consistent with results from other studies [66, 103].

### Amino acid decarboxylases, Na^+^/H^+^ antiporters, K^+^ transporters, and proton-efflux ATPases contribute to habitation across wide pH ranges

**Fig. 6:**
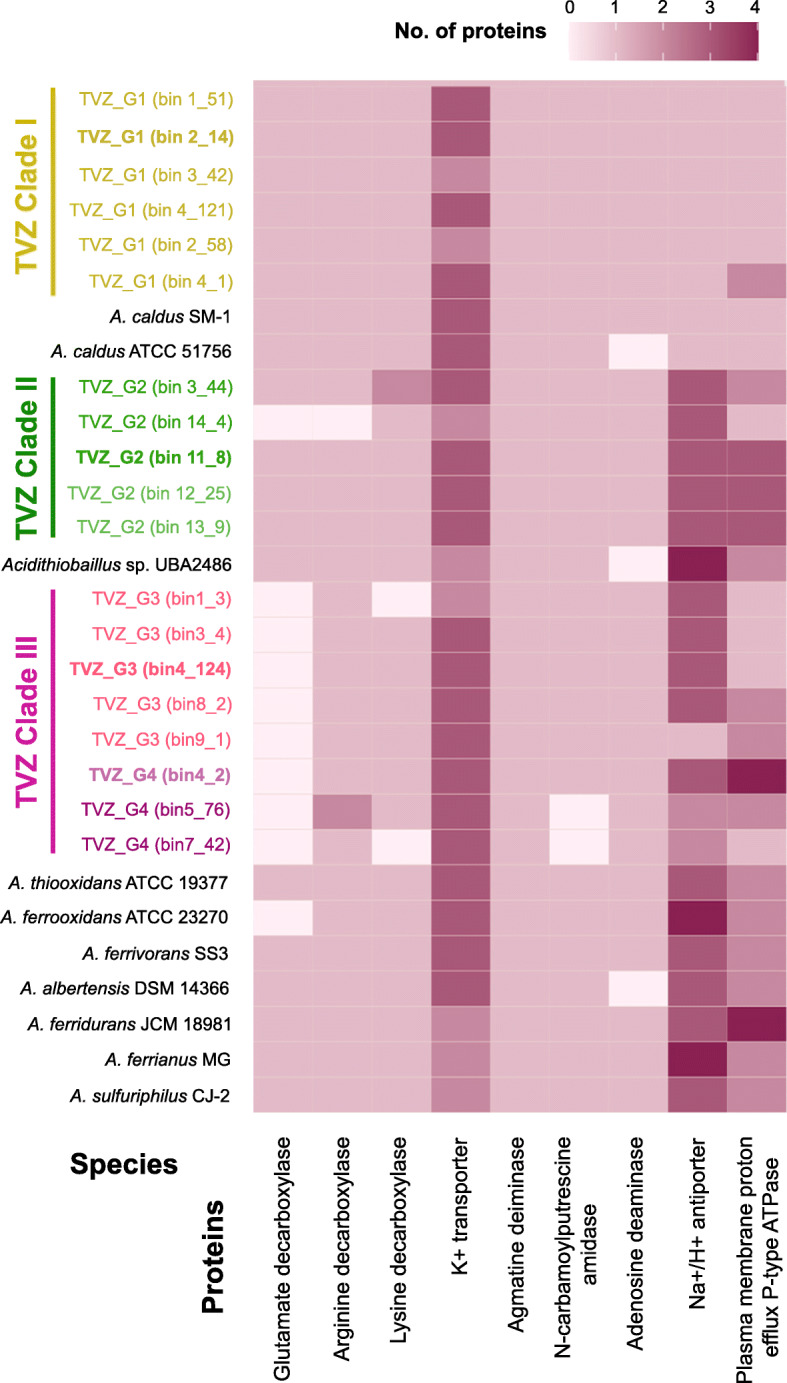
Heatmap showing the number of proteins associated with pH homeostasis in the TVZ and other *Acidithiobacillus* species. Bold script indicates the representative genome of the TVZ *Acidithiobacillus* species. Accession IDs of genes analyzed can be found in Table S7

All four TVZ *Acidithiobacillus* populations were detected across wide pH ranges exceeding the previously known limits of other *Acidithiobacillus* species. Here we examined genes encoding five groups of proteins involved in pH homeostasis: amino acid decarboxylases (using H^+^ in the decarboxylation of amino acids), K^+^ transporters, deiminases/deaminases (generating ammonium ions from ammonia and H^+^), Na^+^/H^+^ antiporters, and plasma membrane proton-efflux P-type ATPases [104–107] (Table S7). Gene prediction results show that all four TVZ populations share similar numbers of gene copies encoding proteins in K^+^ transporters (two to three copies each) and that this is also similar to those found in other *Acidithiobacillus* genomes (Fig. 6). Comparably, we found the TVZ and other *Acidithiobacillus* share one copy each of genes encoding proteins in the deiminases/deaminases group (agmatine deiminase, N-carbamoylputrescine amidase, and adenosine deaminase), with few exceptions (e.g., *A. caldus* ATCC 51756 and *A. albertensis* DSM 14366 lack adenosine deaminase) (Fig. 6). K^+^ uptake helps maintaining pH homeostasis by generating the internal positive membrane potential [104, 108], while deiminase/deaminase reactions create ammonia as a by-product which later combines with protons to produce ammonium ions [106]. Nevertheless, TVZ populations differ in the number of gene copies they respectively have that encode amino acid decarboxylases, Na^+^/H^+^ antiporters, and plasma membrane proton-efflux P-type ATPases.

We predict that TVZ_G1 and other *A. caldus* primarily rely on amino acid decarboxylases, in addition to K^+^ transporters, to maintain pH balances (Fig. 6). They were found to possess one copy each of genes encoding three distinct amino acid decarboxylases, and fewer copies of genes than the other TVZ groups (only one each) for Na^+^/H^+^ antiporter and plasma membrane proton-efflux ATPase. The pH homeostasis genes detected in *A. caldus* TVZ_G1 were similar to those of other *A. caldus* strains. This is likely due to their close phylogeny (Fig. 4). These genes are likely preserved by TVZ_G1, despite genome streamlining, due to their significance for inhabiting acidic environments. Although the amino acid decarboxylation indeed helps in eliminating intracellular protons, whether the decarboxylated product (e.g., GABA from glutamate decarboxylation) is exported from the cells is still being debated. Some studies claim that GABA might be kept in the cells after decarboxylation and can be further transformed and included in the TCA cycle [109, 110]. Other studies suggest that it is exported right after decarboxylation [104–107]. Either way, all 19 TVZ and 10 reference *Acidithiobacillus* genomes studied here were found to lack the genetic capacity to convert GABA into succinate semialdehyde and succinate (via GABA transaminase (EC 2.6.1.19) and succinate-semialdehyde dehydrogenase (EC 1.2.1.16)), and the complete glutamate decarboxylase exporting system was also not identified. Nonetheless, a previous study has shown that amino acid decarboxylation was highly expressed by *A. caldus* when introduced to acid stress [111].

In contrast to the *A. caldus* group, up to three gene copies encoding Na^+^/H^+^ antiporters and another three for plasma membrane proton-efflux ATPases are present in TVZ_G2 MAGs, alongside three more for amino acid decarboxylases. These results suggest TVZ_G2 rely on all three mechanisms to cope with excess protons to maintain their intracellular pH. MAGs in both the closely related TVZ_G3 and TVZ_G4 Clade III groups also tended to possess more than one gene copy encoding Na^+^/H^+^ antiporters (up to three). Strikingly, however, none contained genes for glutamate decarboxylase. In addition, TVZ_G3 MAGs tended to have fewer gene copies than TVZ_G2 for plasma membrane proton-efflux ATPase (one or two) (Fig. 6), while TVZ_G4 MAGs were found to possess up to four plasma membrane proton-efflux ATPase gene copies.

As for TVZ_G2-G4, Na^+^/H^+^ antiporter gene copies were also found to be high, similar to those found in *A. thiooxidans*, *A. ferrooxidans*, *A. ferrivorans*, and *A. albertensis* (Fig. 6); all except for *A. ferrivorans* have wide pH growth ranges (0.5–6.0) (Fig. 3b). This implies that possessing more Na^+^/H^+^ antiporters genes might be a key strategy allowing these *Acidithiobacillus* to live across wide pH ranges. In addition, Na^+^/H^+^ antiporters are driven by proton motive force generated by the respiratory chain, i.e., electrochemical gradients [104, 105]. They, therefore, potentially consume less energy than amino acid decarboxylation (which requires the uptake of amino acids and the exclusion of decarboxylated products) and plasma membrane proton-efflux P-type ATPases (which cost ATP) [104, 105, 112, 113]. As such, the use of antiporters for pH tolerance could also be an energy-efficient mechanism for streamlined microorganisms.

## Conclusions

Four thermoacidophilic populations of *Acidithiobacillus*, which included three novel genome-inferred species and one new *A. caldus* clade, were recovered in this study. Three of these are abundant, cosmopolitan populations, widely distributed across TVZ geothermal environments and extreme ranges of temperature (17.5–92.9 °C) and pH (1.0–7.5); in both cases exceeding the previously known upper physiological limits of *Acidithiobacillus*. Hot spring *Acidithiobacillus* genomes were found to have high GC and proline-coding contents, which are interpreted to increase thermostability, potentially enabling them to live at high temperatures. Moreover, results indicate the hot spring *Acidithiobacillus* have undergone genome streamlining, likely as a result of thermal adaptation. In addition, we predicted that some TVZ *Acidithiobacillus* (TVZ_G1-G2) cope with the broad pH conditions via dependence on a higher number of amino acid decarboxylase genes. TVZ_G2-G4 also shared a roughly similar number of Na^+^/H^+^ antiporters and plasma membrane proton-efflux proteins with other reference *Acidithiobacillus* species that have similarly wide pH ranges, suggesting the importance of these two predicted proteins in organisms inhabiting a broad spectrum of pHs. While experimental confirmation of bioinformatic predictions is needed, results provide insights into genomic features and adaptations that we infer enable hot spring-associated *Acidithiobacillus* species to colonize a broad range of physicochemically diverse environments.

## Supplementary Information

**Additional file 1.**

**Additional file 2.**

## Data Availability

All amplicon and WGS data from sinter samples can be accessed through NCBI under BioProject PRJNA543937. All amplicon and WGS data from sediment samples can be accessed through NCBI Bioproject PRJNA644733.

## Abbreviations

ANI: Average nucleotide identity
ASV: Amplicon sequence variant
CDS: Coding sequence
DDH: DNA-DNA hybridization
GTDB: Genome Taxonomy Database
SNV: Single-nucleotide variant
TVZ: Taupo Volcanic Zone
WGS: Whole genome sequencing
