# Supplementary material for: Genomic adaptations enabling Acidithiobacillus distribution across wide-ranging hot spring temperatures and pHs
Source: Microbiome. 2021 Jun 11;9:135. doi: 10.1186/s40168-021-01090-1 (PMC8196465; doi:10.1186/s40168-021-01090-1)
Supplement: Supplementary file 3 — Additional file 2. [file 40168_2021_1090_MOESM2_ESM.pdf]

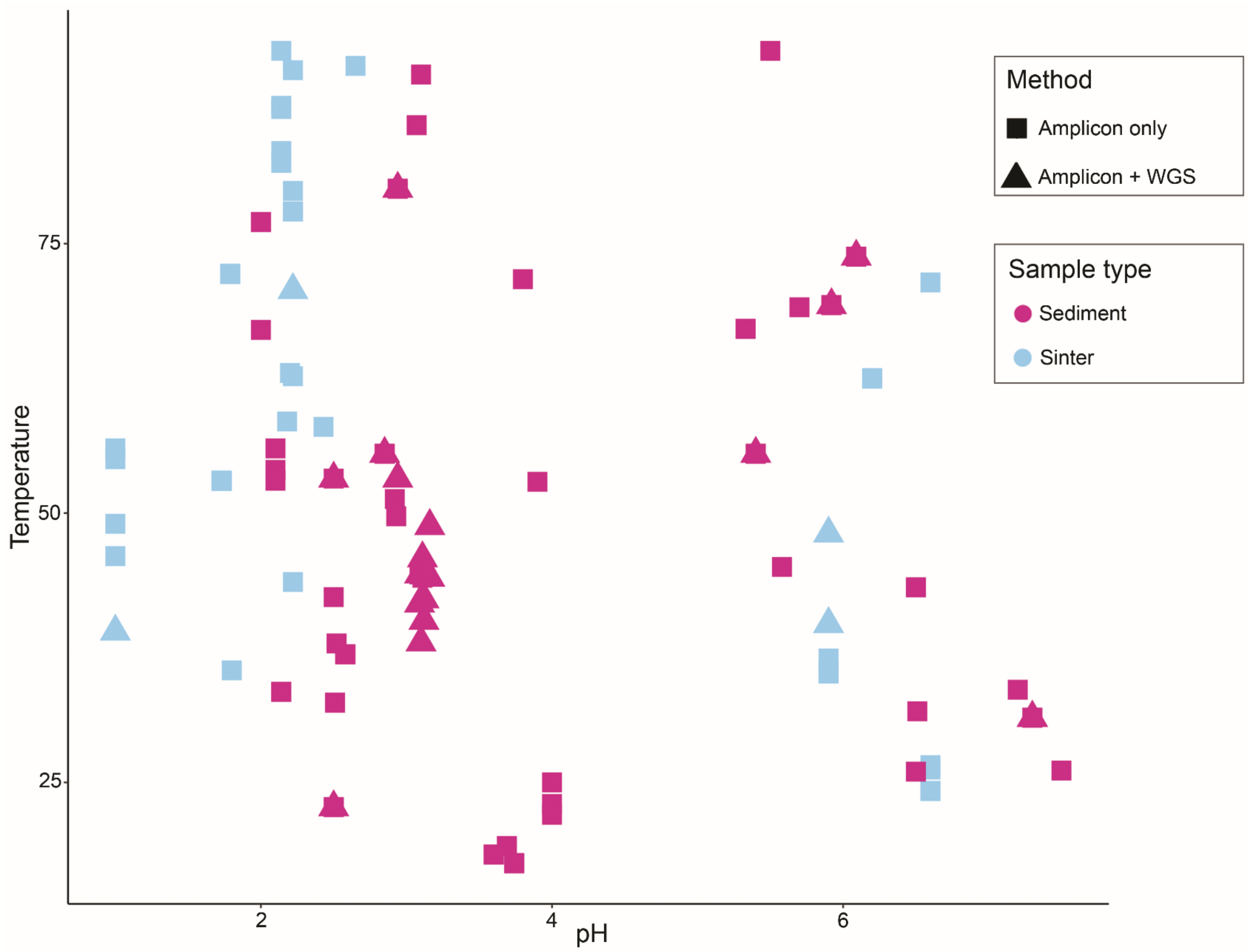

Figure S1. Temperature and pH of all samples collected in this study. Amplicon sequencing was undertaken on all 138 samples (square), and WGS sequencing was undertaken on 22 representative samples (triangle).

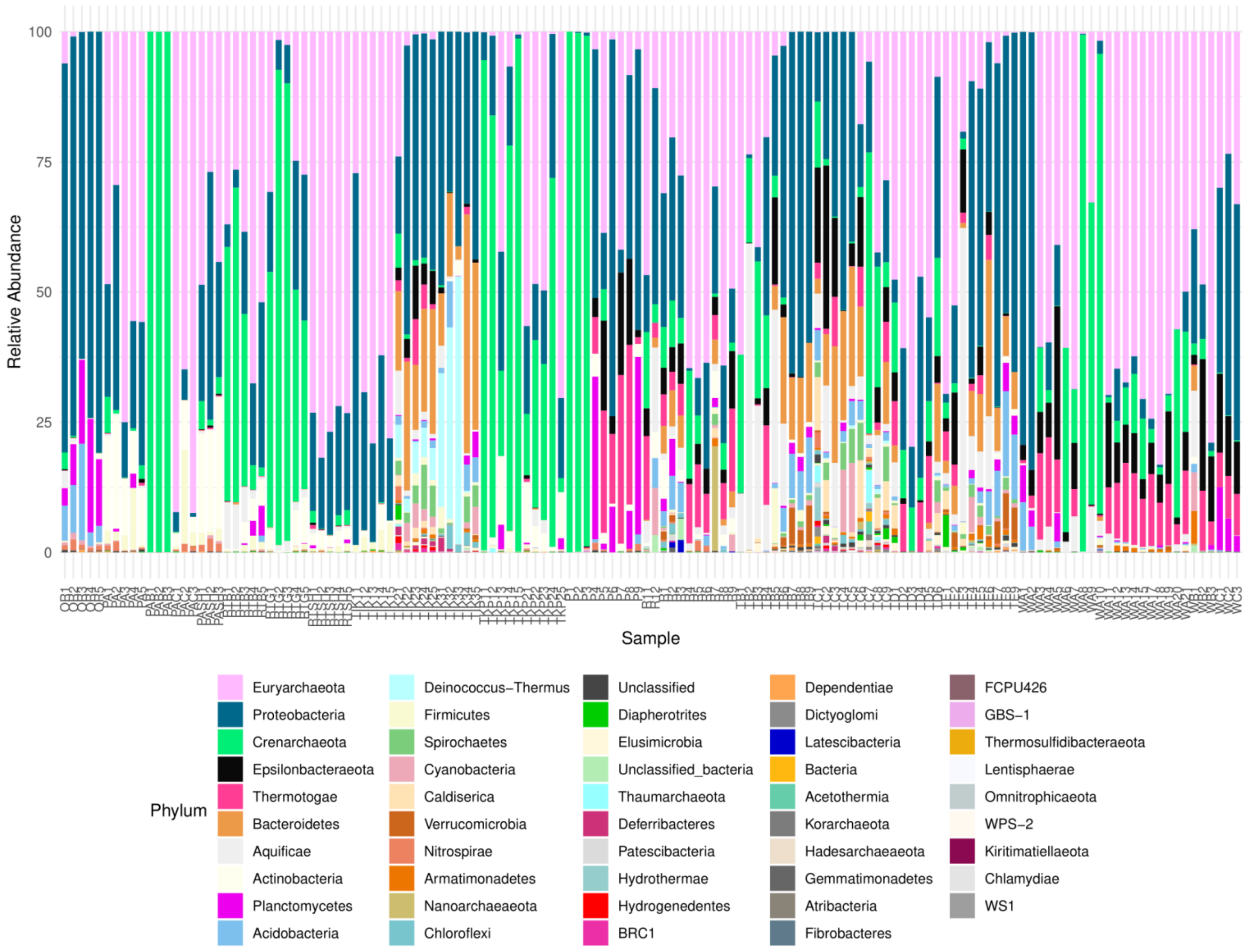

Figure S2. 16S rRNA gene relative abundance of overall prokaryotic communities across all samples.

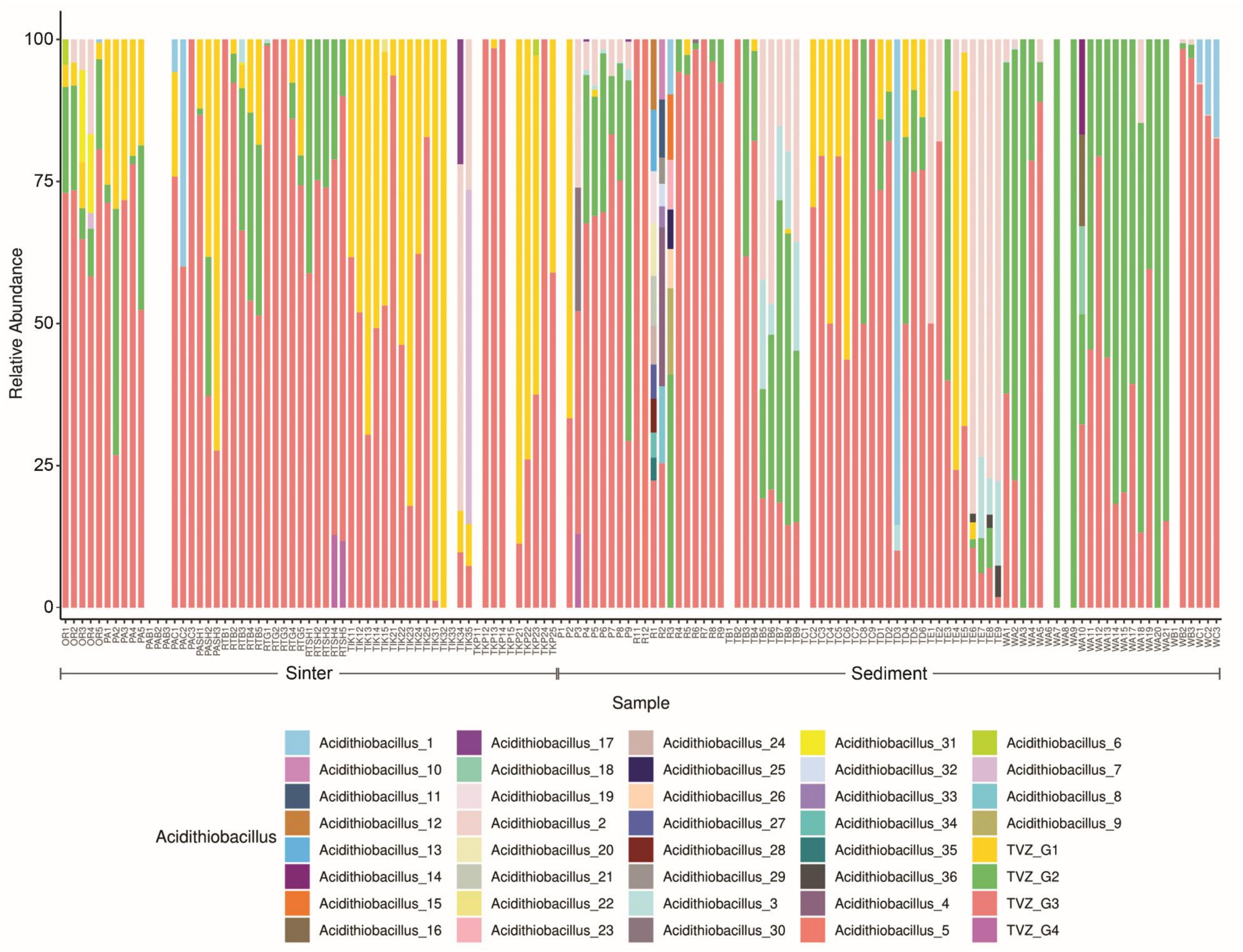

Figure S3. 16S rRNA gene relative abundance of top 40 *Acidithiobacillus* ASVs found in sinter and sediment communities. Sinter data derived from [7].

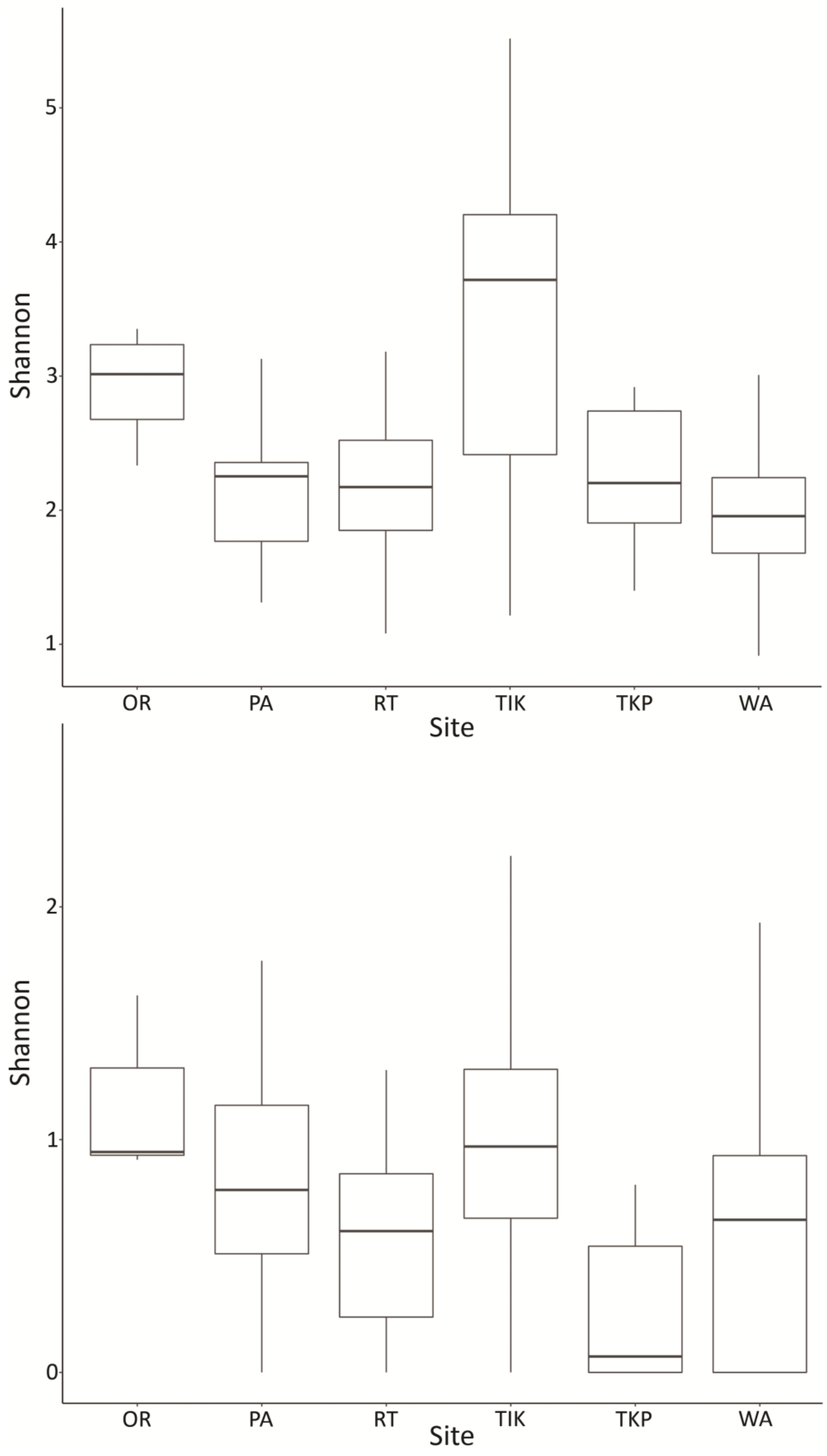

Figure S4. Shannon's diversity of overall community (upper) and *Acidithiobacillus* population (lower) at each sampling site.

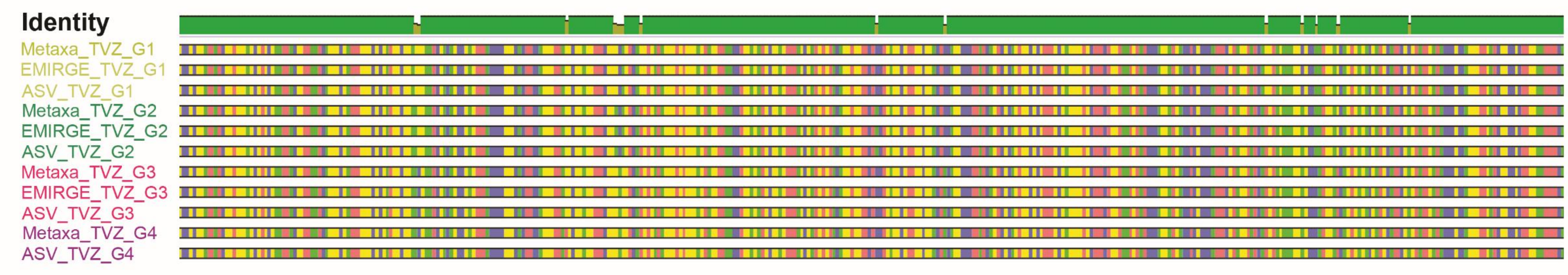

Figure S5. Alignments of partial 16S rRNA sequences (388 bp) of the 4 TVZ *Acidithiobacillus* species derived from MeTaxa2, EMIRGE and amplicons. Note that only MeTaxa2 and amplicon sequences were extracted and derived for TVZ_G4.

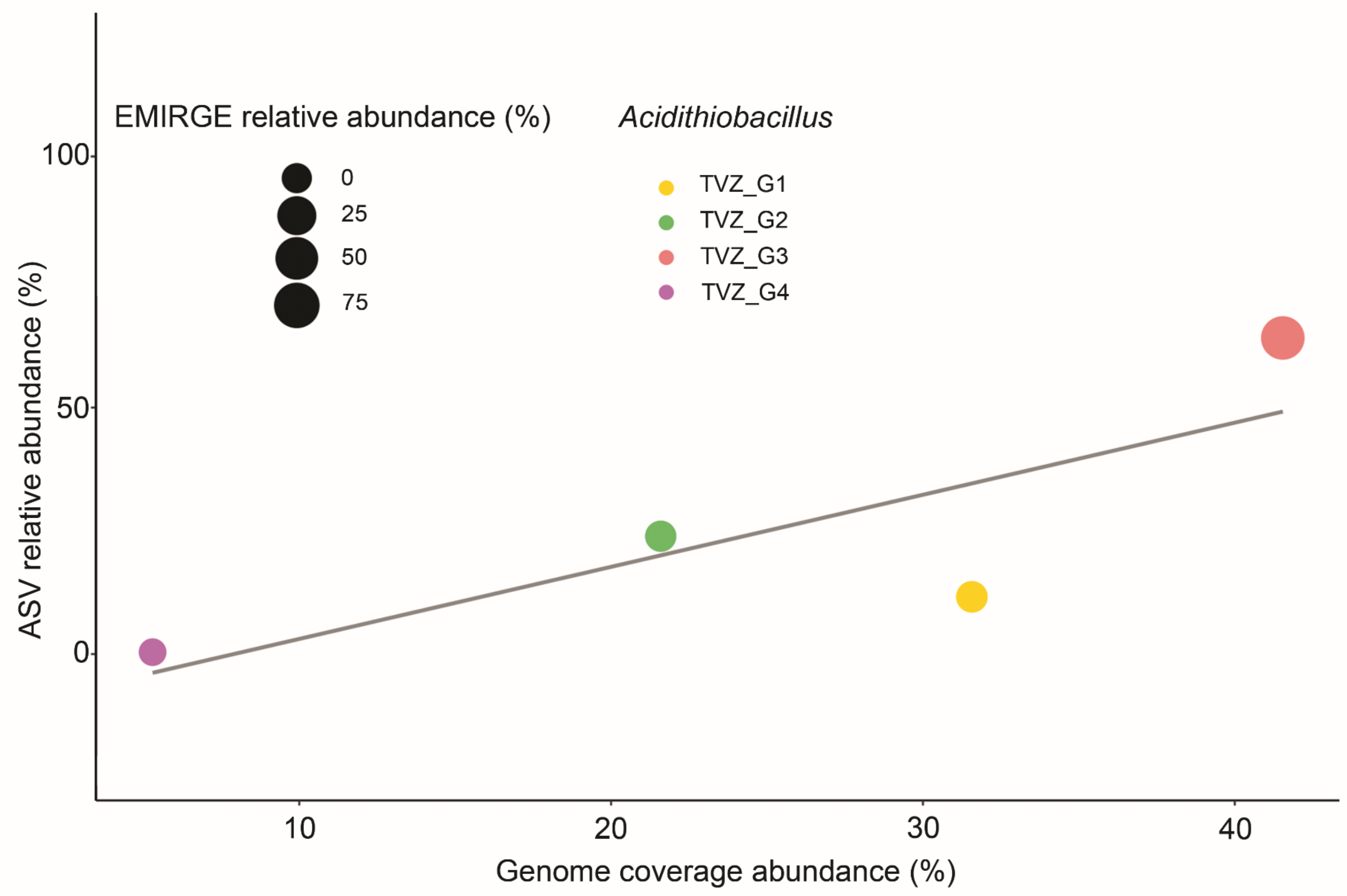

Figure S6. Scatterplot showing correlations between ASV relative abundance (%), genome coverage abundance (%), and EMIRGE relative abundance (%). The abundances of four representative genomes from each genome cluster, four ASVs , and three EMIRGE sequences (except for TVZ_G4 which could not be reconstructed, hence its EMIRGE relative abundance = 0) were compared pairwise to confirm that each of all three were from similar genomes. R = 0.81, *p* = 0.19; Pearson's Correlation Coefficient.

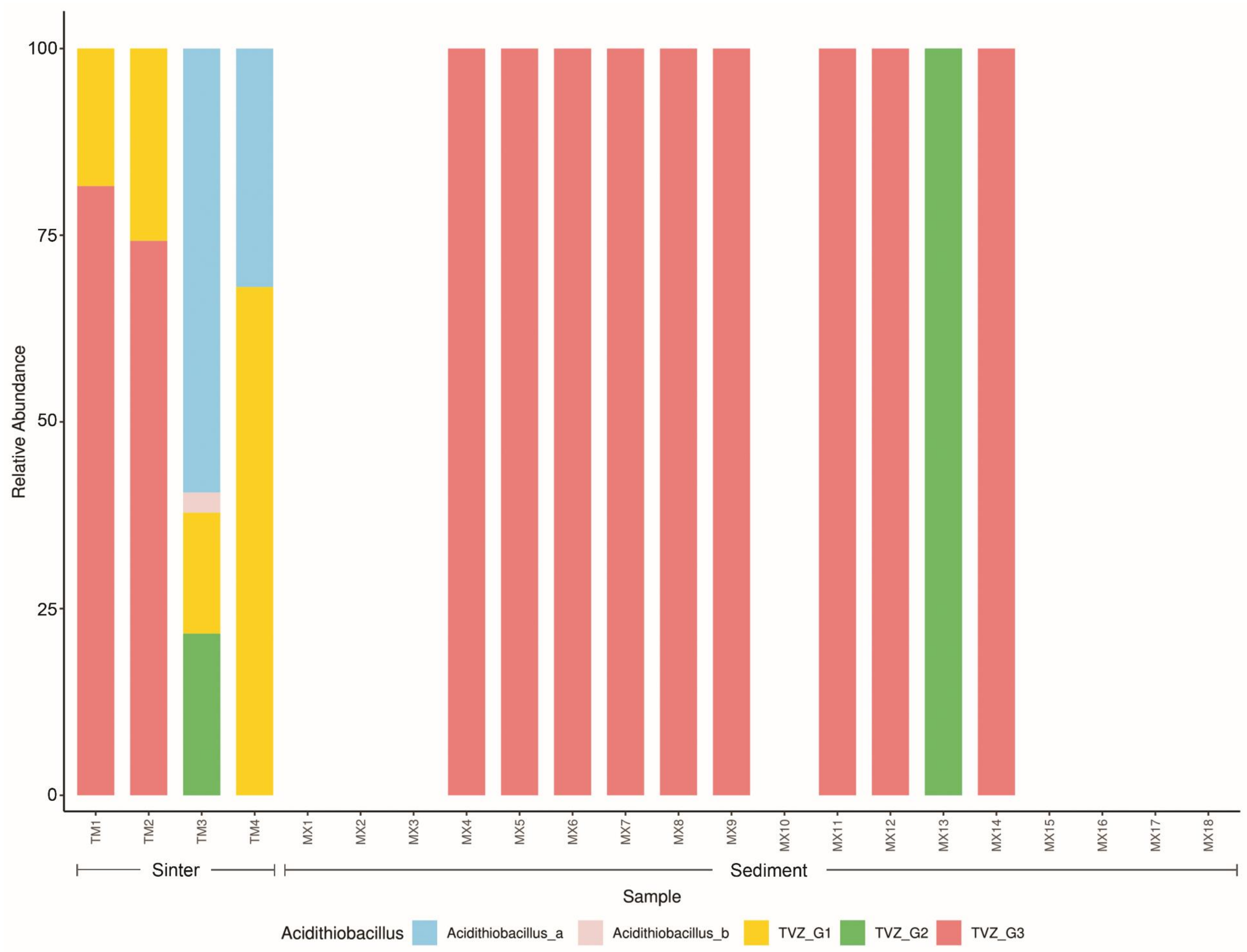

Figure S7. Relative abundance of 16S rRNA gene reconstructed by EMIRGE of *Acidithiobacillus* found in sinter and sediment communities. Sinter data derived from [7]. Keys for WGS sample names are shown in Table S1.

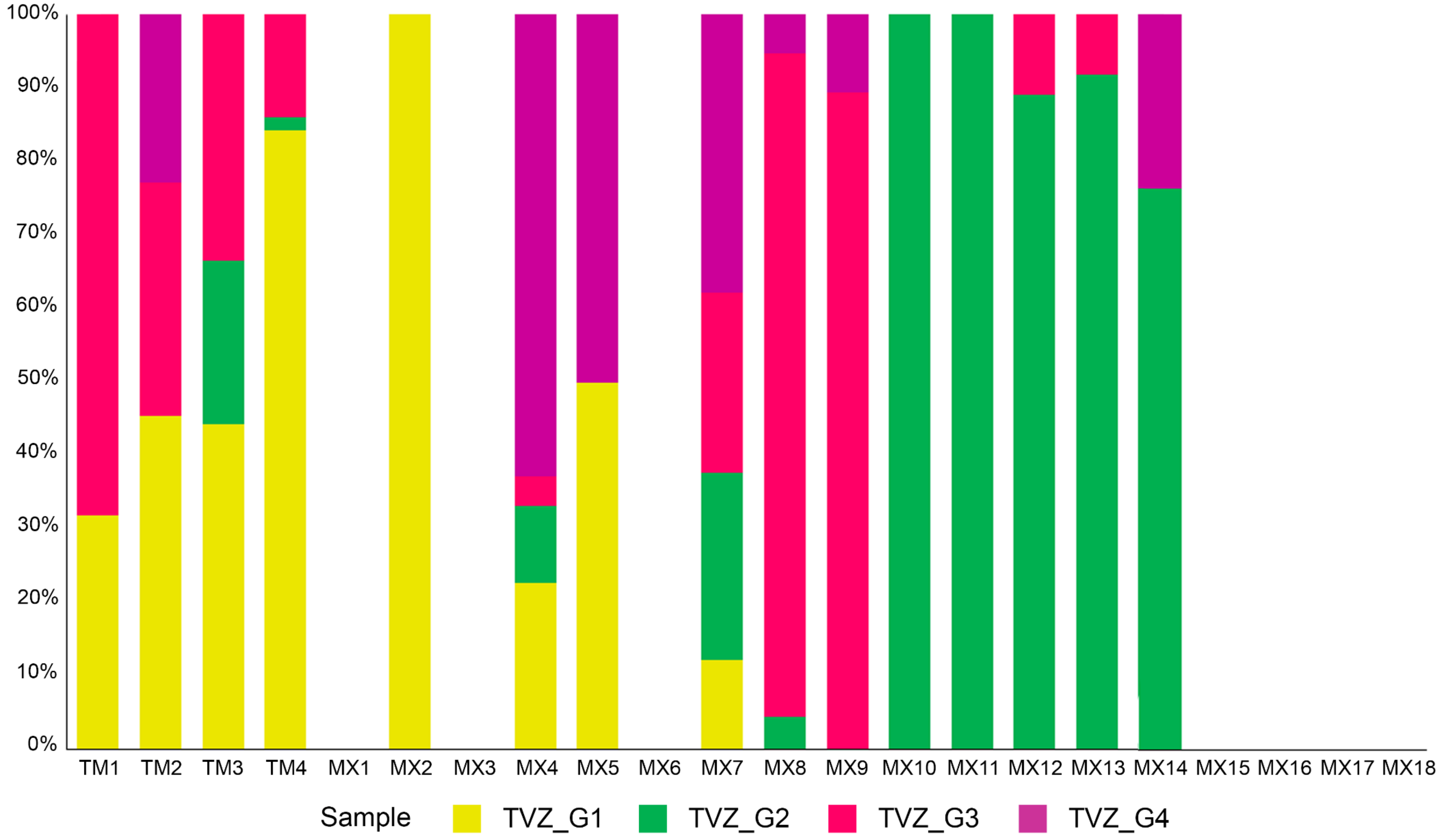

Figure S8. Genome coverage abundance of the TVZ *Acidithiobacilllus* groups across all 22 representative samples. Keys for WGS sample names are shown in Table S1.

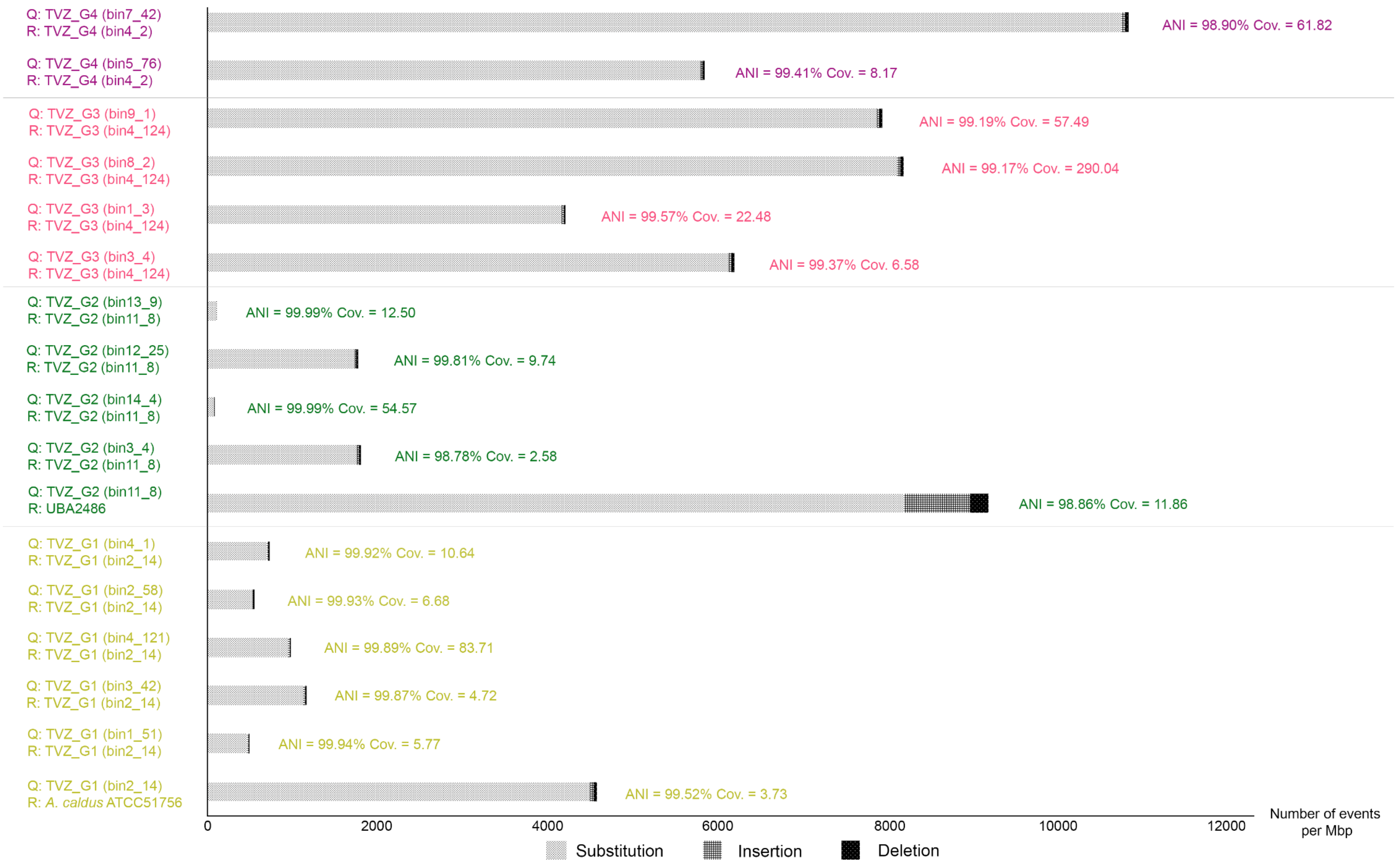

Figure S9. Number of single nucleotide variants (SNVs) substitutions, insertion and deletion events (indels) per Mbp in query genomes (indicated by Q along the y-axis) compared to reference genomes (indicated by R) per TVZ group. Average coverages (Cov.) of query contigs that had SNVs and indels are shown next to bars, along with the ANI between the query and reference genomes.
